# Insulin-like growth factor-I receptor activity is essential for Kaposi's sarcoma growth and survival

**DOI:** 10.1038/sj.bjc.6602408

**Published:** 2005-04-05

**Authors:** S-B Catrina, M Lewitt, C Massambu, A Dricu, J Grünler, M Axelson, P Biberfeld, K Brismar

**Affiliations:** 1 Department of Molecular Medicine, Diabetes Center Karolinska, Karolinska Hospital, M1:02, Karolinska Institutet, Stockholm, Sweden; 2 Immunopathology Lab, Karolinska Hospital, M1:02, Karolinska Institutet, Stockholm, Sweden; 3 Department of Clinical Chemistry, Karolinska Hospital, M1:02, Karolinska Institutet, Stockholm, Sweden

**Keywords:** Kaposi's sarcoma, IGF-I, IGF-II IGF-IR, VEGF, proliferation, growth factors, apoptosis, picropodophyllin

## Abstract

Kaposi's sarcoma (KS) is a highly vascular tumour and is the most common neoplasm associated with human immunodeficiency virus (HIV-1) infection. Growth factors, in particular vascular endothelial growth factor (VEGF), have been shown to play an important role in its development. The role of insulin-like growth factors (IGFs) in the pathophysiology of different tumours led us to evaluate the role of IGF system in KS. The IGF-I receptors (IGF-IR) were identified by immunohistochemistry in biopsies taken from patients with different AIDS/HIV-related KS stages and on KSIMM cells (an established KS-derived cell line). Insulin-like growth factor-I is a growth factor for KSIMM cells with a maximum increase of ^3^H-thymidine incorporation of 130±27.6% (*P*<0.05) similar to that induced by VEGF and with which it is additive (281±13%) (*P*<0.05). Moreover, specific blockade of the receptor (either by *α* IR3 antibody or by picropodophyllin, a recently described selective IGF-IR tyrosine phosphorylation inhibitor) induced KSIMM apoptosis, suggesting that IGF-IR agonists (IGF-I and -II) mediate antiapoptotic signals for these cells. We were able to identify an autocrine loop essential for KSIMM cell survival in which IGF-II is the IGF-IR agonist secreted by the cells. In conclusion, IGF-I pathway inhibition is a promising therapeutical approach for KS tumours.

The development of Kaposi's sarcoma (KS) is characterised by spindle cell (SC) proliferation, inflammatory cell infiltration, neoangiogenesis, oedema and invasiveness. It is the most frequent tumour in AIDS patients and, although antiretroviral therapy seems to improve the prognosis of patients with AIDS-KS tumours, almost all patients eventually develop disseminated disease (Bower *et al*, 1999). The lack of specific therapy is a driving force towards the identification of possible new therapeutic targets.

Kaposi's sarcoma origin and pathogenesis is still debated. Along with infectious agents such as HIV-1 and HHV-8 (also known as KS-associated herpes virus) (the subject of a recent review (Boshoff and Weiss, 2002)), cytokines and growth factors appear to play an important role in KS development (Ensoli and Sturzl, 1998; Skobe *et al*, 1999). Vascular endothelial growth factor (VEGF) is suggested to have a central paracrine/autocrine role for tumour growth (Masood *et al*, 1997). However, blocking VEGF with a specific antibody seems to affect mainly the vascularisation of the tumour rather than the tumour cell behaviour itself (Samaniego *et al*, 2002). Therefore, a specific alternative approach, which would interfere with tumour proliferation and survival, could be of value for KS therapy. KS cells display not only a dysregulation of the proliferation, but they are also resistant to apopotosis (Mori *et al*, 1996; Buttiglieri *et al*, 2004a). Insulin-like growth factor-I (IGF-I) is an important mitogen and antiapoptotic factor in various tissues. It can act as a systemic hormone or as a local growth factor. The systemic levels of IGF-I are positively influenced mainly by growth hormone (GH) and by nutrition (Yakar *et al*, 2002). The biological effects of IGF-I are mediated through the activation of IGF-I receptors (IGF-IR) which exhibit the same affinity for IGF-II and can also be activated by high doses of insulin (LeRoith and Roberts Jr, 2003). Experimental and epidemiological evidence highlights the involvement of IGF-I system in different malignancies (Khandwala *et al*, 2000), as well as an essential role in angiogenesis along with VEGF (Smith *et al*, 1999). Interference with IGF-IR function in several neoplastic diseases seems to be an attractive pharmaceutical approach especially nowadays when specific and easy-to-deliver compounds are available (LeRoith and Helman, 2004). To our knowledge, no previous studies have addressed the role of IGF-I on KS biology.

In this study, we have used a KS cell line (KSIMM) to evaluate the importance of IGF-I on KS biology. These cells produce highly angiogenic and haemorrhagic tumours when injected in nude mice, mimicking the human disease (Albini *et al*, 1997). We make the novel observation that IGF-I receptors (IGF-IR) are essential for both proliferation and antiapoptotic signals. Moreover, we identified an autocrine loop essential for KSIMM cell survival, in which IGF-II is the tumoral ligand for IGF-IR. The presence of IGF-IR on the surface of AIDS-KS tumour cells *in situ* suggests that our experimental observation can be used as a basis for the pharmacological IGF-IR interference for the treatment of patients with this disease. In this respect, a novel specific IGF-IR tyrosine phosphorylation blocker, picropodophyllin (PPP), which has shown antitumoral properties *in vivo* (Girnita *et al*, 2004), induced apoptosis in KSIMM cells.

## MATERIALS AND METHODS

### Materials

Recombinant human IGF-I was provided by Pharmacia (Stockholm, Sweden), IGF-II was a kind gift from Eli Lily (Indianapolis), VEGF-165 was purchased from R&D Systems (Abingdon, UK) and human insulin from Novo Nordisk (Denmark). Cell culture reagents were from Gibco (Stockholm, Sweden), ^3^H-thymidine was purchased from NEN Life Sciences Products and scintillation liquid (Ultima Gold) from Perkin-Elmer (Boston, USA). Mouse monoclonal antibody against the *α* subunit of IGF-IR (2C8), used for immunohistochemistry, and mouse monoclonal-anti-CD34 (endothelial antigen) were from Santa Cruz Biotechnology, Inc. (USA); anti-mouse IgG and peroxidase-linked whole antibody were from Amersham (Uppsala, Sweden), and mouse monoclonal anti-IGF-IR antibody (*α*IR 3), used for cell culture, was obtained from Oncogene Research Products (Boston, USA). Picropodophyllin was prepared as described previously (Girnita *et al*, 2004). All other reagents used for immunohistochemistry were from DAKO (Denmark).

### Immunohistochemistry for detecting IGF-IR and CD34 expression

Diagnostic, surgical biopsies of AIDS-related KS lesions from patients in Tanzania (Dept. Pathology, Muhimbili Medical School, Dar El. Salaam) (*n*=8, four nodular and four patch lesions) were formalin-fixed, embedded in paraffin and sectioned for histopathological and immunohistochemical evaluation. The patients' HIV status was evaluated by serology using the Wellcozyme Recombinant anti-HIV-1 ELISA VK56 (Murex Diagnostics, Toronto, Canada). Serial tissue sections were evaluated for IGF-IR and CD34 expression using ABC immunohistochemistry with an anti-avidin enhancement technique (van Gijlswijk *et al*, 1996). Paraffin sections were deparaffinised, rehydrated and pretreated by microwave heating in citrate buffer, pH 6, and endogenous peroxidase activity blocked by hydrogen peroxide (3% for 30 min), as described previously (Kaaya *et al*, 2000). After rinsing and blocking with normal horse serum, serial sections were incubated with anti-IGF-IR or anti-CD34 antibody for 2 h at 37°C. This was followed by rinsing and incubation with biotinylated secondary antibody (horse anti-mouse) for 40 min and another rinsing and incubation with ABC peroxidase (30 min). To increase the sensitivity of ABC, a biotinylated antibody against avidin (30 min) and a second ABC peroxidase treatment (30 min) was performed. Bound ABC was visualised by incubation with fresh 3,3′-diamino benzidine (DAB) for 2–10 min. Negative controls using isotype-matched antibody were included and placenta biopsies were used as IGF-IR positive control.

Insulin-like growth factor-I receptor immunostaining in KSIMM cells cultured on chamber slides and fixed in paraformaldehyde (PFA) 4% in PBS, pH 7.4, was performed using the monoclonal mouse IgG1 antibody at a concentration of 10 *μ*g ml^−1^ as described previously (Catrina *et al*, 2002). Negative controls using isotype-matched IgG were included.

### Cell culture

KSIMM cells, kindly provided by Dr A Albini (Istituto Nazionale per la Ricerca sul Cancro, Genova, Italy), were cultured in DMEM supplemented with 2 mM L-glutamine, 100 IU ml^−1^ penicillin and streptomycin and 10% heat-inactivated bovine serum (FBS) in a humidified atmosphere with 5% CO_2_ at 37°C. Proliferation experiments and MTT assay experiments were carried out in 96-well microtest plates (plated at a density of 1 × 10^4^ cells well^−1^) with five replicates for each concentration, for FACS analysis in 100 mm Petri dishes and for TdT-mediated dUTP nick end labelling (TUNEL) assay in chamber slides (Nalge Nunc, Naperville, USA) with duplicates. The experiments were repeated at least three times. At 24 h following plating, the cells were rinsed once with medium without FBS, serum-starved for 24 h, rinsed again with medium without FBS and incubated with testing substances for the times indicated, as volumes less than 0.01%.

### Evaluation of cell proliferation by ^3^H-thymidine incorporation assay

After 44 h of incubation with testing substances, 1 *μ*Ci ^3^H-thymidine was added to each well. After 4 h, the cells were washed twice with 0.9% ice-cold NaCl. The cell-associated radioactivity, precipitated with 5% TCA, was determined by liquid scintillation counting. Results are expressed as a percentage of the control, untreated cells.

### MTT assay

After 44 h of incubation with PPP or control (DMSO), the number of viable cells was evaluated using the MTT viability assay, as we described previously (Catrina *et al*, 1999). Briefly, 20 *μ*l MTT (3-[4, 5-dimethylthiazol-2-yl]-2, 5-diphenyltetrazolium bromide; thyazolil blue) (5 mg ml^−1^) in DMEM was added to each well. After 4 h of incubation, the formazan crystals, produced by viable cells, were dissolved with 100 *μ*l 0.004 N HCl-isopropyl alcohol for 5 min. The cell survival rate was calculated from the optical density at 570 nm after subtracting the optical density at 620 nm. Results are expressed as a percentage of the control treated cells.

### Evaluation of cell apoptosis

#### TdT-mediated dUTP nick end labelling assay and morphological evaluation

TdT-mediated dUTP nick end labelling was carried out using a fluorescein-labelled *in situ* cell death detection kit (Roche, Bromma, Sweden) as described previously (Catrina *et al*, 2002). Briefly, the cells fixed in PFA were permeabilised with 0.1% Triton X-100 in 0.1% sodium citrate solution for 2 min on ice and 50 *μ*l TUNEL reaction reagent/sample was applied over the cells. After 60 min incubation at 37°C in the dark, the slides were examined by fluorescence microscopy. For light microscopy evaluation, the slides were incubated for 30 min, 37°C with 50 *μ*l converter POD in PBS 0.1% BSA, and exposed to DAB solution (0.5 mg ml^−1^) (Sigma). Finally, the samples were counterstained with Meyer haematoxylin. The classical morphological apoptosis criteria of nuclear condensation, membrane blebbing and formation of apoptotic bodies combined with TUNEL-positive reaction of the nuclei were used to evaluate, by light microscopy, the apoptotis, which was expressed as an apoptotic index (percentage of apoptotic cells from 500 counted cells).

### FACS with annexin V/propidium iodide

Following incubation with specified substances, cells were trypsinised and then stained with annexin V and propidium iodide as specified by the manufacturer (R&D Systems, Oxon, UK) and analysed by flow cytometry. In order to compare the effect of different substances on the survival rate, we generated histograms for annexin V fluorescence, and positive cells were gated and expressed as percentages from the total number of acquired cells.

### Insulin-like growth factor-I and IGF-II assay in KSIMM cell culture medium

Insulin-like growth factor-I and IGF-II in conditioned KSIMM cultured medium were assayed after HPLC separation from IGFBPs by radioimmunoassay (RIA) using high-affinity antibodies. The IGF-I assay was previously described (Bang *et al*, 1991) and has a detection limit 0.07 ng ml^−1^ with intra- and interassay coefficients of variation 4 and 11%. Respectively, IGF-II assay was performed under the same conditions as IGF-I assay, except that IGF-II labelled by the chloramine T method was used as a tracer and an anti-IGF-II mouse monoclonal antibody (S1F2) was used as first antibody in a final concentration of 1 : 150 000 (Upstate Biotechnology, Lake Placid, NY, USA), and the separation was performed using an anti-mouse IgG (Sac-Cel, IDS Ltd, Boldon, UK). The detection limit was 0.05 ng ml^−1^, with an intra-assay coefficient of variation of 3%.

The HPLC separation of the IGFs from IGFBPs was performed using a size-exclusion chromatography after incubation for 30 min at 22°C of 50 *μ*l conditioned culture medium with 50 *μ*l column buffer (5 ×) (acetic acid 0.2 M, trimethylamine 0.1 M and Triton X-100 0.5 g l^−1^, pH 2.8 adjusted with sulphuric acid) as described previously (Crawford *et al*, 1992).

### Statistical analysis

All values are presented as mean±s.e. The data were analysed by one-way ANOVA with a Tukey *post hoc* test; or Kruskall–Wallis one-way analysis of variance with Dunn's method, as appropriate, using SigmaStat v2.03 SPSS Inc.

## RESULTS

### Insulin-like growth factor-I receptors are expressed in AIDS-related KS tumours

**Figure 1:**
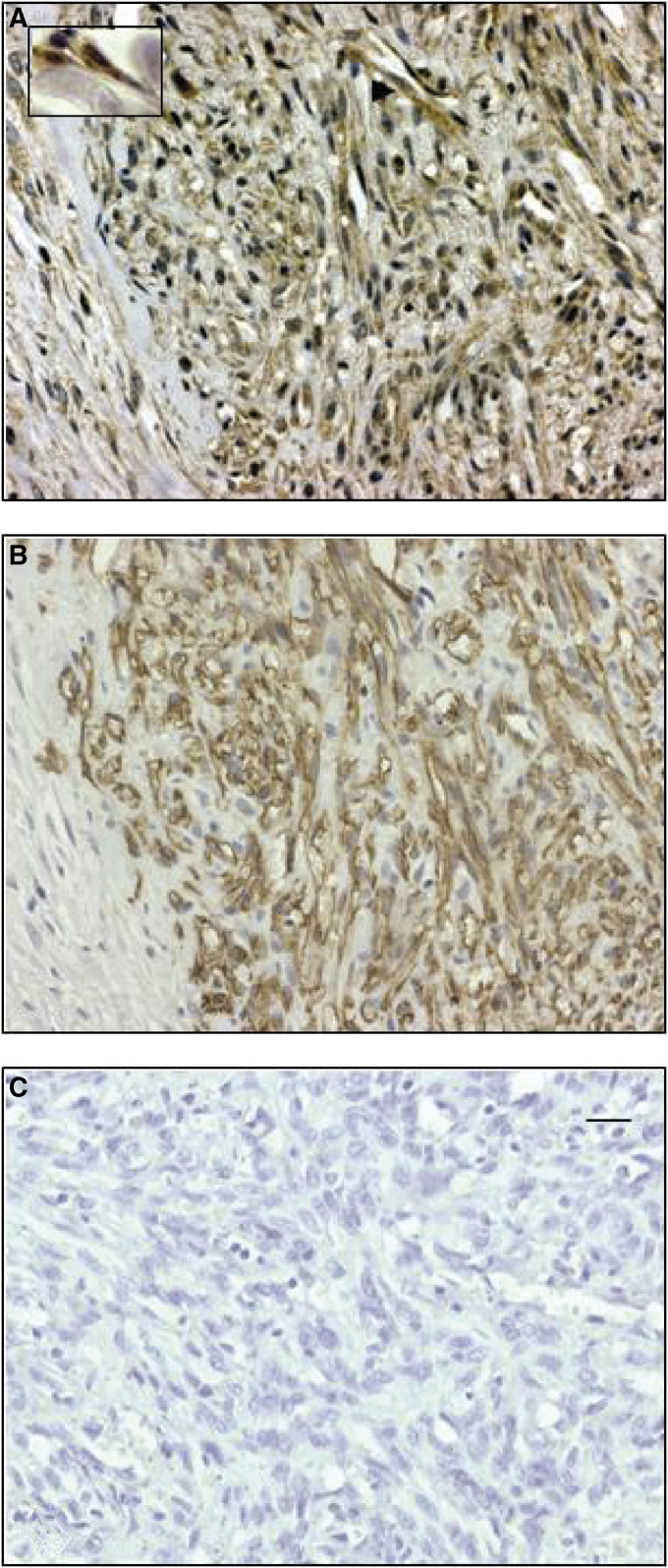
AIDS-KS biopsies show immunoreactivity for IGF-IR. Serial sections of paraffin-embedded KS biopsies were evaluated for IGF-IR and CD34 expression. (**A**) Immunohistochemistry for IGF-IR showing reactivity in tumour SCs (small arrow) and some infiltrating leukocytes (long arrow). Original magnification × 250. The inset panel shows IGF-IR reactivity in spindle tumour cells at a higher magnification (× 500). (**B**) Immunohistochemistry for CD34 showing reactivity in tumour SCs. (**C**) Negative control with mouse IgG1.The bar represents 15 *μ*m.

The expression of IGF-IR was evaluated in eight cases of AIDS-related KS tumour biopsies by immunohistochemistry. A large proportion of the KS tumour SCs, identified by morphology and positive immunostaining for CD34 in serial sections, showed positive staining with the anti-IGF-IR antibody. In addition, most vessels and slit-lining cells, and some infiltrating leucocytes, were positively stained (Figure 1A, B). No significant difference was seen between patch (early) and nodular (late) lesions in the immunostaining pattern (data not shown).

### Insulin-like growth factor-I stimulates KSIMM cell proliferation

**Figure 2:**
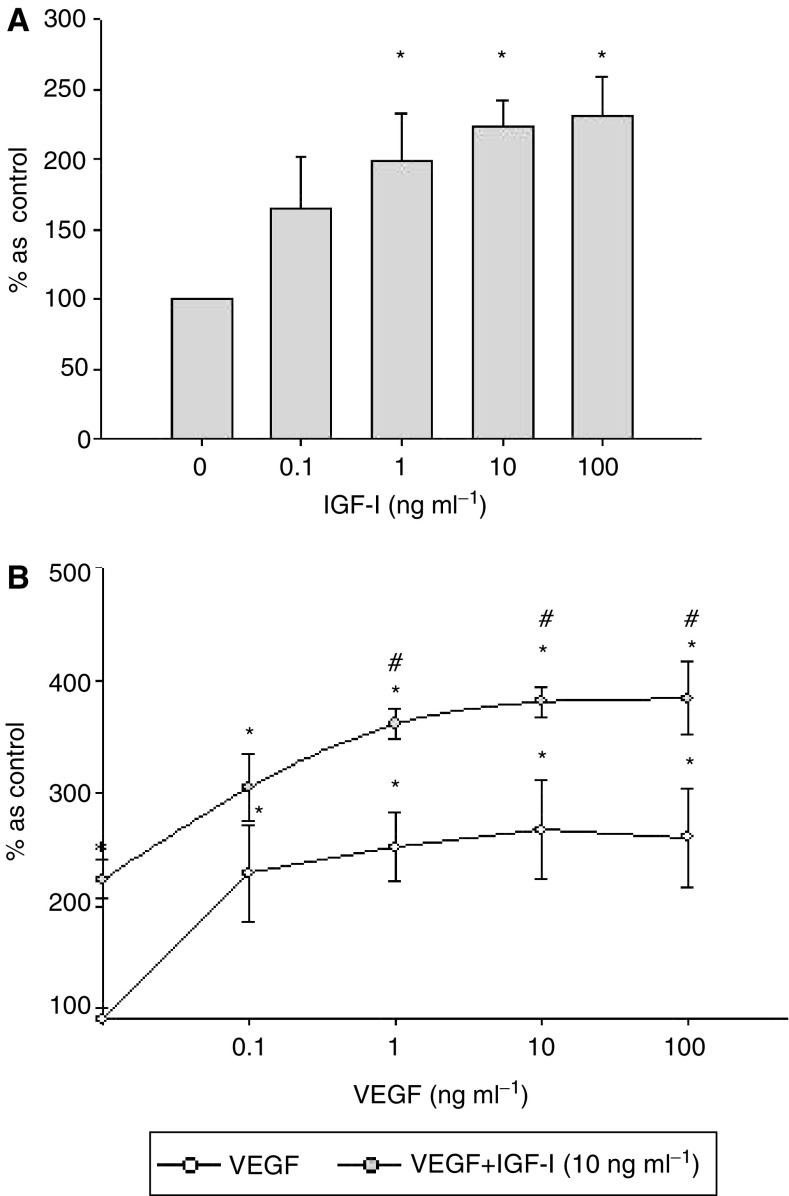
Insulin-like growth factor-I increases in a dose-dependent manner proliferation of KSIMM cells with potency similar to VEGF, with which it has an additive effect. (**A**) KSIMM cells, starved for 24 h, were exposed to either IGF-I or vehicle for 48 h when proliferation was assessed by ^3^H-thymidine incorporation. The values represent means±s.e.m. from five different experiments (^*^P<0.05). (**B**) KSIMM-starved cells were exposed to either VEGF alone (0.1–100 ng ml^−1^) or to the combination of VEGF and 10 ng ml^−1^ IGF-I. Cell proliferation was assessed by ^3^H-thymidine incorporation after 48 h incubation. The values represent means±s.e.m. from three different experiments (^*^P<0.05 as control, ^#^*P*<0.05 as equivalent VEGF concentration).

The effect of IGF-I on the proliferation of KSIMM cells was investigated using the ^3^H-thymidine incorporation. Insulin-like growth factor-I stimulated the proliferation of KSIMM cells *in vitro* in a dose-dependent manner by a maximum increase of 130±27.6% (*P*<0.05 for concentrations higher than 1 ng ml^−1^) (Figure 2A). The effect of IGF-I on the proliferation of KSIMM cells was of the same magnitude as VEGF, postulated to be an essential growth factor for KS cells (maximum increase of 151.9±30.5%; *P*<0.05 for concentrations higher than 1 ng ml^−1^) (Figure 1B). The combination of 10 ng ml^−1^ of IGF-I (maximally effective stimulatory concentration) and VEGF (0.1–100 ng ml^−1^) had an additive effect, increasing the proliferation rate by a maximum of 281±13% (*P*<0.05) (Figure 1B).

### The growth-promoting effects of IGF-I on KSIMM cells are mediated by IGF-IR

**Figure 3:**
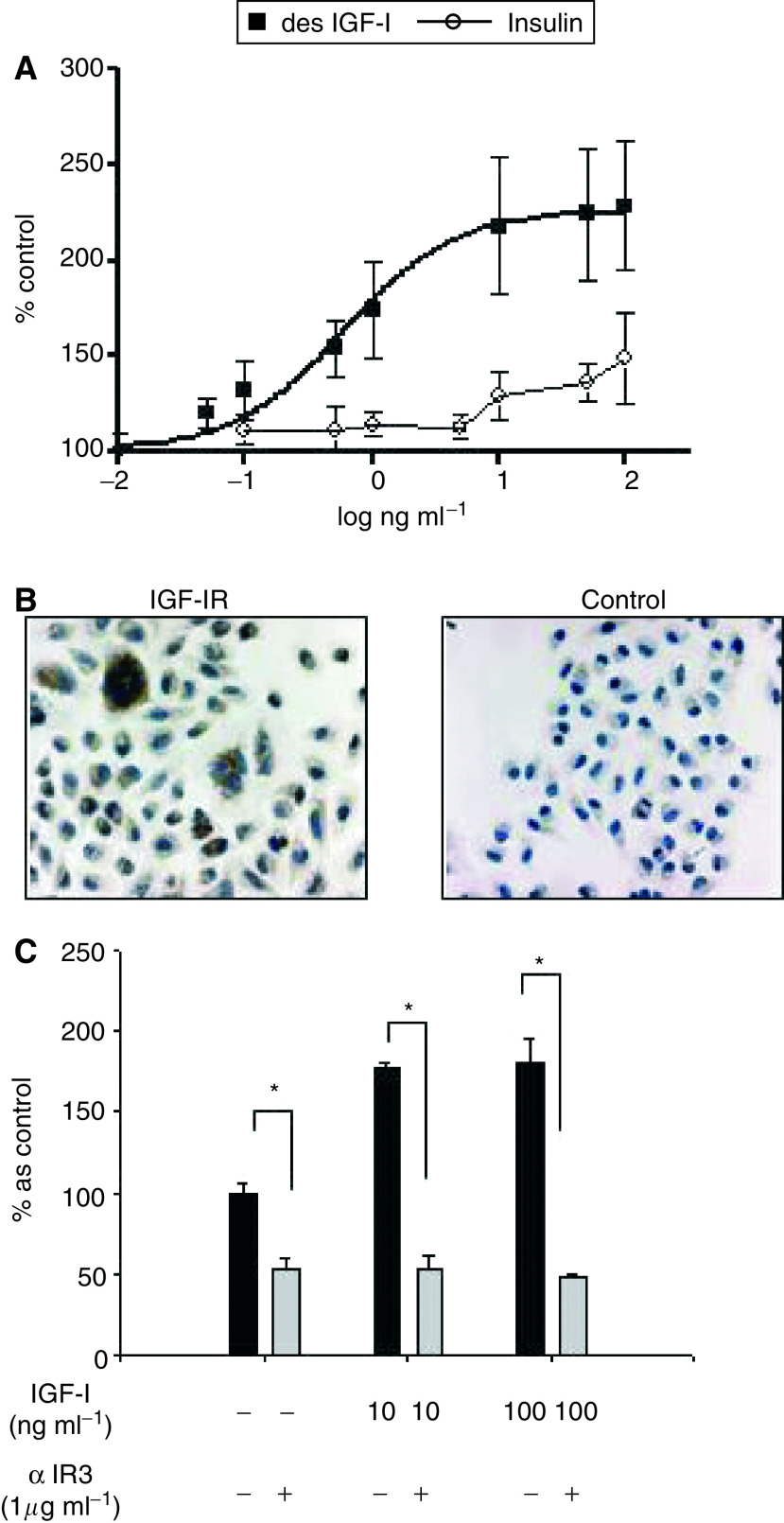
Insulin-like growth factor-I growth-promoting effect on KSIMM cells is mediated by IGF-IR. (**A**) KSIMM cells, starved for 24 h, were exposed to either des(1–3) IGF-I or insulin for 48 h when proliferation was assessed by ^3^H-thymidine incorporation. The values represent means±s.e.m. from three different experiments. (**B**) Immunohistochemical identification of IGF-IR in KSIMM. The left panel shows intra and pericellular positivity of the KSIMM cells for IGF-IR. The right panel shows the negative control with mouse IgG1. (**C**) KSIMM cells, starved for 24 h, were exposed to IGF-I alone (10 and 100 ng ml^−1^) or in combination with the specific blocking antibody for IGF-IR, *α* IR3. After 48 h, proliferation was assessed by ^3^H-thymidine incorporation. The values represent means±s.e.m. from three experiments (^*^P<0.05).

To investigate what receptor mediates the effect of IGF-I, we have compared the growth-promoting effect of des (1–3) IGF-I (agonist for IGF-IR, which binds minimally to the IGFBPs) with insulin. Des (1–3) IGF-I stimulates the growth rate of KSIMM cells in a dose–response manner with an EC_50_ around 0.58 ng ml^−1^ far more potent than insulin that, at 100 ng ml^−1^, reaches less then 50% of the maximal effect of IGF-I. This suggests that IGF-IR is the mediator of IGF-I effects on KSIMM cells (Figure 3A). The presence of this receptor in KSIMM cells was further demonstrated by immunohistochemistry. As presented in Figure 3B, KSIMM cells are specifically immunopositive for the *α* subunit of IGF-IR with both cytoplasmic and pericellular patterns. The functional involvement of these receptors as mediators of the IGF-I growth-promoting effect was demonstrated by the complete abolishment of the IGF-I effect when the cells were co-incubated with *α* IR3 monoclonal blocking antibody (Flier *et al*, 1986) (Figure 3C).

### Insulin-like growth factor-I receptor mediates antiapoptotic signals in KSIMM cells

**Figure 4:**
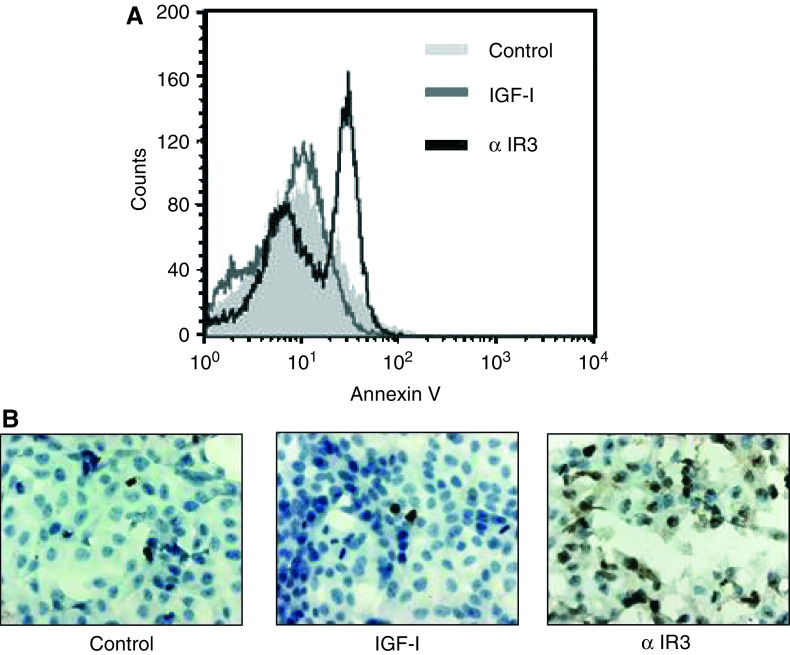
Insulin-like growth factor-I receptor mediates antiapoptotic signals for KSIMM cells. (**A**) KSIMM cells were treated, after starving for 24 h, with either IGF-I (100 ng ml^−1^), *α* IR3 (1 mg ml^−1^) or vehicle for 24 h and analysed by FACS for annexin V binding. (**B**) KSIMM cells were starved for 24 h and then treated with either IGF-I (100 ng ml^−1^), *α* IR3 (1 *μ*g ml^−1^) or vehicle for 48 h, fixed in PFA 4% and then stained for TUNEL and counterstained with Meyer haematoxylin (magnification × 500).

The profound inhibition of ^3^H-thymidine incorporation in KSIMM cells after specific blockade of the IGF-IR prompted us to investigate a potential antiapoptotic effect mediated through IGF-IR. Treatment of the cells with IGF-I had no significant effect on the apopotic rate of KSIMM cells (apoptotic index 1.3±0.3 *vs* 3.8±0.7% in control), but blocking IGF-IR with *α* IR3 monoclonal antibody induced apoptosis. This effect was documented both by the positivity of KSIMM cells for Annexin V (Figure 4A) and by typical apoptotic morphology and TUNEL-positive staining (Figure 4B) after treatment with *α* IR3 (apoptotic index 21.8±0.7 *vs* 3.8±0.7% in control) (*P*<0.001). No changes in the number of propidium iodide cells were observed following treatment, confirming the specific proapoptotic effect of IGF-IR blocking.

### Insulin-like growth factor-II is the endogenous agonist of an autocrine loop in KSIMM cells

Specific blocking of IGF-IR resulted in decreased spontaneous ^3^H-thymidine incorporation, as well as induction of apoptosis, suggesting the presence of an autocrine loop essential for both proliferation and survival of these tumoral cells. Thus, we measured the putative ligands for IGF-IR (IGF-I and IGF-II) in KSIMM conditioned media after separation from IGFBPs. Insulin-like growth factor-II has been detected in 72 h conditioned media at a concentration of 4.1 ng ml^−1^, while IGF-I was under the detection limit of our assay. As expected, exogenous IGF-II had the same growth-promoting effect as IGF-I on KSIMM cells, with a maximum ^3^H-thymidine incorporation of 157±21% (*P*<0.05).

### Picropodophyllin, a specific inhibitor of IGF-IR activity, induces apoptosis in KSIMM cells

**Figure 5:**
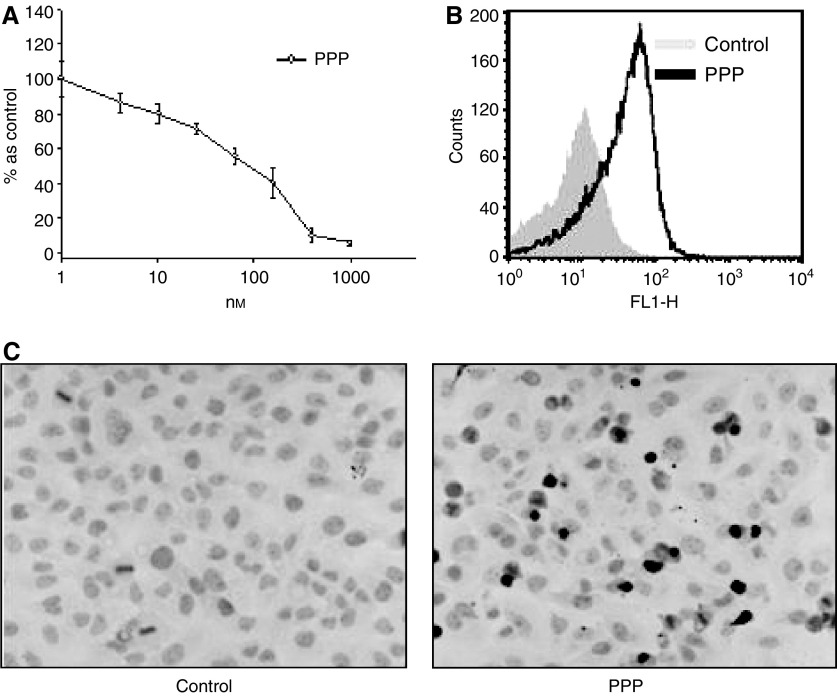
Picropodophyllin, a specific inhibitor of IGF-IR activity, induces apoptosis in KSIMM cells. (**A**) KSIMM cells were treated with different concentrations of PPP for 48 h and subjected to MTT assay for the last 4 h. (**B**) KSIMM cells were treated, after starving for 24 h, with PPP (1 *μ*g ml^−1^) or vehicle for 24 h and analysed by FACS for annexin V binding. (**C**) KSIMM cells were starved for 24 h and then treated with PPP (1 mg ml^−1^) or vehicle for 48 h, fixed in PFA 4% and then stained for TUNEL and counterstained with Meyer haematoxylin (magnification × 500).

The presence of IGF-IR in the tumour specimens from patients with AIDS-KS coupled with the essential functional role of the IGF-IR in KSIMM biology determined us to investigate the effect of PPP, a new, specific IGF-IR tyrosine kinase inhibitor, which has been shown to have antitumoral proprieties *in vivo* (Girnita *et al*, 2004). Treatment of KSIMM cells with PPP induced a dose-dependent cell death with an IC_50_ at 95 nM (Figure 5A), in the range reported for other IGF-IR-positive tumours (Girnita *et al*, 2004). Treatment of KSIMM cells with 100 nM PPP induced apoptosis as documented by Annexin V positivity (Figure 5B) and by typical apoptotic morphological cell appearance and positive immunostaining for TUNEL (Figure 5C) (22.1±1.5 *vs* 1.3±0.5% in control-treated cells) (*P*<0.001).

## DISCUSSION

KS is the leading cause of cancer death in areas with endemic AIDS (Parkin *et al*, 1999). Even though highly active antiretroviral therapy (HAART) has led to a decrease of the KS tumour burden, AIDS patients are still at high risk of developing KS when they discontinue HAART or when the therapy is not available. Developing a therapy specific to the tumour cell biology is imperative for these patients.

Interference with growth factors of importance in KS biology is a logical approach which has already been successful in different malignancies (for a review, see Shawver *et al*, 2002). Here, we show the presence of the IGF-IR in biopsies taken from patients with AIDS/HIV-related KS, and further demonstrate that the IGF system is essential for cell growth and for mediating antiapoptotic signals in a KS cell line (KSIMM) that has the characteristics of the KS SCs and produce large highly vascularised tumours when injected s.c. in nude mice (Albini *et al*, 1997). Moreover, we suggest that PPP, a small molecule that specifically interferes with IGF-IR function, is a potential effective therapy.

Insulin-like growth factor-I receptor was found in all the KS tumours studied. No clear difference in IGF-IR cell expression between early and late KS stages was observed, suggesting a constant IGF-IR expression during characteristic multistage development of KS tumours.

The relevance of the functional role of these receptors in KS biology was further demonstrated in KSIMM cells where exogenous IGF-I induced a dose-dependent stimulation of cell proliferation. Insulin-like growth factor-I has been shown to be a growth factor for different tumours and prospective epidemiological data suggested that high circulating levels of IGF-I confer increased risk for different solid tumours (reviewed by LeRoith and Roberts Jr, 2003). For KSIMM cells, IGF-I had the same growth-promoting potency as VEGF, which is postulated to be one of the most important growth factors for KS tumours (Arasteh and Hannah, 2000). Furthermore, IGF-I and VEGF have an additive effect on the KSIMM proliferation rate, which suggests that these two growth factors may act at least in part independently to promote growth. Vascular endothelial growth factor and IGF-I cellular pathways have been reported to either act independently (Hellstrom *et al*, 2001) or to interact (Smith *et al*, 1999) in normal endothelial cells. The relationship between VEGF and IGF-I signalling pathways including those involved in cell survival in KS is the focus of ongoing studies.

Both IGF-IR ligands (IGF-I and IGF-II) have high affinity for IGFBPs, molecules that can compete with their binding to the receptor. Thus, to investigate the potential receptor involved in IGF effect on KSIMM cells, we have used des(1–3)-IGF-I, a full agonist for IGF-IR that binds minimally to IGFBPs (Clemmons *et al*, 1992).

The effect of IGF-I on KSIMM cells is mediated through IGF-IR. This is suggested by the higher potency of des(1–3)-IGF-I as compared to insulin, as well as the complete abolition of the IGF-I effect through IGF-IR-blocking antibody (*α* IR3). Moreover, we demonstrated that both KSIMM cells as well as KS tumours express IGF-1R. Even though hybrid receptors with insulin cannot be excluded (Pandini *et al*, 2002), the effect of PPP (specific blocker of the beta subunit of IGF-IR (Girnita *et al*, 2004)) on KSIMM apoptosis suggests that the IGF-IR is the main mediator of IGF system on these cells. The decrease of ^3^H-thymidine incorporation in KSIMM cells after treatment with the IGF-IR-blocking antibody *α* IR3 powerfully suggested the presence of an autocrine loop in these cells. We have in consequence analysed the secretion of both IGF-I and IGF-II by KSIMM cells because both are equally potent ligands for these receptors. Both IGF-I and IGF-II mRNA expression have been documented previously in AIDS-KS cultured cells (Weich *et al*, 1991) but, to our knowledge, there is no previous report on their secretion in KS cells. To avoid the known interferences of IGFBPs with IGF-I and IGF-II assays, which could give false-positive results, we have first separated them by HPLC. We were able to detect IGF-II but not IGF-I in the conditioned medium. The presence of the IGF-II as member of the endogenous IGF system is not surprising. There is clear evidence of its upregulation in different tumours (Zhang *et al*, 1997).

We were able to show that the autocrine loop plays an essential role for protecting the KSIMM from apoptosis. This was demonstrated by blocking the IGF-IR in two ways: by using *α* IR3 (a specific blocking antibody) or by using PPP (specific blocker of the beta subunit of IGF-IR (Girnita *et al*, 2004)). Exposure of the KSIMM cells to either of these compounds induced apoptosis as demonstrated by both externalisation of phosphatidylserine, TUNEL staining and typical apoptotic morphological changes. We did not observe any significant difference in the apoptosis rate after treatment with IGF-I, which is in line with the reported low spontaneous apoptosis rate of these cells (Buttiglieri *et al*, 2004b).

In addition to autocrine sources, it may be that KS tumours are stimulated by endocrine IGFs from the circulation. Albini *et al* (1999) made the observation that the *in vivo* administration of somatostatin decreases the development of KS-like lesions in mice inoculated with KSIMM cells, despite an absence of somatostatin receptors on the tumoral cell surface. In the light of our data on the effect of IGF-I in these cells, we speculate that somatostatin, by inhibiting pituitary GH release, may also have had an antitumoral effect via a decrease in endocrine IGF-I, as it was demonstrated for other tumors (Wu *et al*, 2003).

Numerous attempts have been made to block the IGF system for treatment of tumours (reviewed by Surmacz, 2003) but most of the methods had different problems as lack of specificity, difficulty of drug delivery, etc. One of the most promising approach is to use specific small molecules that inhibit IGF-IR tyrosine kinase (LeRoith and Helman, 2004). In this light, we have also tested the effect of PPP, which has been shown to be a potent and specific inhibitor of the IGF-IR tyrosine kinase (Girnita *et al*, 2004). Treatment of the KSIMM cells with PPP induced a dose-dependent apoptosis in the same dose range reported for other IGF-IR-positive cells. We would like to point out that the compound is active even in the presence of FCS, which shows that blocking the IGF system overrides the survival signals from other factors. Specifically inhibiting the IGF-IR tyrosine kinase has been proved to be a successful approach for other small molecules as well (Garcia-Echeverria *et al*, 2004; Mitsiades *et al*, 2004; Warshamana-Greene *et al*, 2004).

In conclusion, we have demonstrated that IGF system is essential for proliferation and survival in a KS cell line, which has characteristics similar to the human disease and we suggest that targeting the IGF-IR could represent a powerful candidate for KS therapy.
